# Methodological Approach for Dengue Viral Load Quantification in Wastewater: Protocol for a Systematic Review and Meta-Analysis

**DOI:** 10.2196/71635

**Published:** 2025-11-13

**Authors:** Nurul Amalina Khairul Hasni, Sakshaleni Rajendiran, Nurul Athirah Naserrudin, Nurul Farehah Shahrir, Terence Yew Chin Tan, Janice Sue Wen Chan, Siti Aishah Rashid

**Affiliations:** 1 Environmental Health Research Center, Institute for Medical Research Malaysia, National Institutes of Health, No. 1, Jalan Setia Murni U13/52, Seksyen U13, Setia Alam, Shah Alam, 40170, Malaysia, 60 0333627443 ext 7443; 2 Institute for Health Systems Research, National Institutes of Health, Shah Alam, Malaysia; 3 Herbal Medicine Research Center, Institute for Medical Research Malaysia, National Institutes of Health, Shah Alam, Malaysia

**Keywords:** Dengue virus, wastewater surveillance, viral concentration, serotypes, sampling methodology

## Abstract

**Background:**

In recent years, the rapid emergence and global spread of dengue has become a public health burden. Clinical surveillance alone has limited capacity, with delayed detection of upcoming outbreaks. Hence, the potential use of wastewater-based surveillance (WBS) for early detection of incoming surges of dengue cases could complement proactive public health action. However, there are still substantial gaps in the standard approach for sampling and detection methods in dengue WBS.

**Objective:**

This review aims to determine the current methodological approach for the detection of dengue virus (DENV) in wastewater across geographical areas.

**Methods:**

The review will be conducted systematically following the PRISMA (Preferred Reporting Items for Systematic Reviews and Meta-Analyses) guidelines. In the initial stage, peer-reviewed publications from PubMed, Embase, Scopus, and Web of Science will be searched using predefined terms such as “Dengue” and “WBS.” Keywords will be adjusted to suit each database to identify studies related to DENV WBS from inception until June 2025. Subsequently, the references from relevant articles will be screened for eligibility. All data will be extracted from full-text articles highlighting the characteristics and methodological context of the investigated DENV WBS using a standardized form. The ROBINS-I (Risk of Bias in Non-randomized Studies - of Interventions) tool and the GRADE (Grading of Recommendations Assessment, Development, and Evaluations) system will be used to assess study bias and the quality of the evidence. Further descriptive analysis and meta-analysis will be applied to evaluate the methodologies for DENV WBS.

**Results:**

The data on DENV detection in wastewater will be synthesized by analyzing sampling techniques, viral detection method, study sites, geographic locations and dengue serotype. A meta-analysis will be conducted using a random-effects model if data are heterogeneous, with pooled estimates reported as 95% CIs. Heterogeneity will be assessed using *I*² and chi-square tests, with subgroup and sensitivity analyses conducted as needed. Findings will be reported in accordance with PRISMA 2020. As of October 2025, records have been identified from the databases and data analysis is expected to be completed by the first quarter of 2026.

**Conclusions:**

This protocol outlines a systematic approach to identifying and evaluating existing methods for detecting DENV in wastewater. This research aims to provide valuable insights into best practices for dengue surveillance and offer guidance for future research by highlighting current strengths and limitations in the field.

## Introduction

Over the past decade, the persistent threat of dengue virus (DENV) has emerged as a significant global health concern, despite the decline in the incidence of other infectious diseases [1,2]. The increasing spread of dengue, an arthropod-borne viral infection, has led to endemic and epidemic transmission cycles in many regions globally. DENV, an arbovirus belonging to the genus *Flavivirus*, has four serotypes ranging from DENV-1 to DENV-4 [2]. The disease is exacerbated by increasing urbanization, increased population density, inadequate housing, improper waste management, scarce water supplies, and climate change [3]. Moreover, the shift and expansion of vector distribution areas and pathogen suitability zones has led to an increase in arboviruses such as DENV [4,5].

Southeast Asia and the Western Pacific region are the hyperendemic hot spots for DENV infections, contributing to nearly 75% of the global dengue burden [6]. The highest number of dengue cases was recorded in 2023, affecting over 80 countries in all World Health Organization regions [6]. Sustained transmission combined with an unexpected rise in dengue infection led to more than 6.5 million cases with more than 7300 dengue-related deaths worldwide [6]. This underscores the critical role of vector-borne disease surveillance, which is an essential part of the dengue control program in many endemic countries [7].

Furthermore, the number of asymptomatic infections was estimated to be higher than those with clinical manifestations, posing a great challenge to public health, considering the former are about 80% as infectious as their symptomatic counterparts [8,9]. The asymptomatic infections may account for 84% of all dengue transmissions, with only 1% attributable to people with clinical manifestations and a positive diagnostic test [9]. Hence, the limitations of clinical and vector surveillance in detecting these subclinical cases may hamper the progress in containing the spread of dengue [10]. Moreover, shifts in circulating DENV serotypes have been associated with the emergence of outbreaks and severe dengue cases, leading to increased morbidity and mortality [11]. This underscores the need for a comprehensive, population-based surveillance system to support timely public health responses and preparedness efforts.

Considering the existing limitations of current dengue surveillance, wastewater-based surveillance (WBS) has been proposed as a promising tool for population-level surveillance [10,12-14]. In addition, DENV RNA has been detected in the urine, rectal swabs, and bodily fluids of infected persons, thereby confirming its presence in the sewage system and reinforcing the applicability of WBS for dengue monitoring [15-17]. Even in areas with low case prevalence and lower shedding, DENV RNA has been successfully detected in wastewater in several studies [10,14]. Additionally, several arbovirus WBS reviews offer valuable insights with focuses on technical feasibility and methods [18,19], highlighting real-world challenges, especially for vector-borne diseases in tropical settings [20]. This review aims to consolidate dengue-specific knowledge.

Despite its potential, the relative importance of dengue WBS has not yet been comprehensively studied. The use of WBS to detect DENV in low-prevalence areas depends heavily on the sensitivity of viral RNA detection in sewage. Accuracy is influenced by factors like population shedding, sewer network characteristics, sampling methods, and analysis techniques. Variability in methodology including sampling strategies, RNA stability, and detection tools (eg, quantitative polymerase chain reaction, sequencing) limits data comparability. The lack of standardized protocols and uneven lab capacity further hinders consistent reporting [21]. A comprehensive evaluation of optimal methods is still lacking, and addressing these gaps is key to improving the reliability and impact of dengue WBS.

Therefore, this review will focus on the methodologies and findings and will highlight the gaps that need to be addressed. Hence, the objectives of this review are to (1) describe methodologies for sampling, detection, and serotype analysis of DENV in wastewater across geographical areas, (2) determine the dengue viral load in different sampling and detection methodologies, and (3) identify potential use of dengue WBS for public health action.

## Methods

### Ethical Considerations

This systematic review has been registered with the National Medical Research Register, Ministry of Health Malaysia, and the protocol has been registered with PROSPERO (International Prospective Register of Systematic Reviews; CRD42024574930). The findings will be disseminated to relevant stakeholders involved in policymaking and program management for dengue surveillance. These results will also help in our future research on dengue wastewater surveillance to choose the best sampling and analysis methods. This review involves data extracted from previously published, publicly available sources and does not directly involve participants or the use of any identifiable personal data. Therefore, formal ethics approval was not required.

This protocol was prepared based on the Preferred Reporting Items for Systematic Review and Meta-Analysis Protocols (PRISMA-P) checklist (Checklist 1). This review is still ongoing and the procedures outlined in this section will be applied during the review process. The systematic review and meta-analysis will be reported in accordance with PRISMA (Preferred Reporting Items for Systematic Reviews and Meta-Analyses) guidelines [22].

### Population

This review will include sewage samples from wastewater treatment plants serving population-based communities or public/private institutions.

### Intervention

We will include studies conducting WBS involving different sampling methodologies (sample type, sampling techniques, frequency, location), detection and serotype analysis.

### Comparator

There is no restriction of the included articles with or without a comparator.

### Outcomes

The outcomes that will be included are viral load (gene copies, Ct value). The outcomes will be compared based on different contexts, which are sampling methodologies and detection methods (polymerase chain reaction–based, enzyme-linked immunosorbent assay) and geographical area.

In addition, we will include studies reporting serotype analysis, positivity rate (%), epidemiological trends (above or below the limits; stagnation, increase, or decrease in the number of cases), and molecular characterization of DENV.

### Patient and Public Involvement

There is no involvement of patients and the public in this review.

### Eligibility Criteria

The eligibility criteria are as described in Textbox 1.

Textbox 1. **Study inclusion and exclusion criteria.**

**Inclusion criteria**

- Preprints and peer-reviewed studies that are descriptive, experimental, cohort, cross-sectional, or case-control, which determine the amount of dengue RNA in wastewater
- Articles published in the English language

**Exclusion criteria**

- Nonoriginal or nonresearch publications (eg, review papers, case reports, technical reports, conference proceedings, books, book chapters, non–full text articles)
- Non-English articles

### Information Source

A comprehensive search will be performed using 4 databases (PubMed, Scopus, Web of Science, and Embase) by two authors independently based on the eligibility criteria. The search strategy will include a combination of predefined keywords that are Medical Subject Headings (MeSH) terms and free-text terms related to dengue WBS. Boolean operators such as OR and AND will be used to combine these keywords and refine the search findings in the titles and abstracts of databases. The search strategy to be used is presented in Checklist 1. Additionally, a manual screening of references from articles will be performed. The time frame for the inclusion of studies will be from inception until June 2025.

### Data Management

Articles retrieved from the electronic searches will be imported into Zotero software (version 6.0.37; Corporation for Digital Scholarship) to check for and remove duplicates. Subsequently, the records will be exported to a CSV file format. Unique identifiers will be given to the records and the data will subsequently be managed through Microsoft Excel (Microsoft Corp).

### Selection of Studies

Preliminary title and abstract screening of the remaining articles will be done independently by two authors to retrieve relevant articles based on the predetermined criteria of the study population, intervention, comparator, and outcomes. Subsequently, full-text articles will be reviewed by two reviewers to assess their eligibility based on the exclusion and inclusion criteria using an eligibility form. If discordance to accept or reject an article arises, a third reviewer will be sought to come to a decision. Reasons for exclusion of articles will also be recorded and discussed. Two independent authors will identify articles to be included in the meta-analysis. The flow of the strategy used for the selection of the included articles will be summarized in a PRISMA diagram as a figure.

### Data Extraction

Comprehensive data extraction will be performed using a standardized form to systematically gather information from each study. This will include study characteristics (author details, publication year, country, study design), sampling methodology (sample type, sampling techniques, frequency, location), method of detection, and serotype analysis. To ensure the reliability of the data, two independent reviewers will perform the extraction, with any discrepancies resolved through discussion or consultation with a third reviewer. This will facilitate a thorough analysis of the evidence, emphasize significant findings, identify research gaps, and establish a basis for future studies in the field.

### Quality Assessment and Risk of Bias

Quality and risk of bias will be appraised with design-specific instruments. The Risk of Bias in Non-randomized Studies – of Interventions (ROBINS-I V2) tool will be used to assess the quality of included studies. This tool evaluates the risk of bias across seven domains: (1) bias due to confounding, (2) bias in the classification of interventions, (3) bias in the selection of participants into the study (or into the analysis), (4) bias due to deviations from intended interventions, (5) bias due to missing data, (6) bias arising from measurement of the outcome, and (7) bias in the selection of the reported result [23]. Each domain will be rated as having a low, moderate, serious, or critical risk of bias [24]. Two independent reviewers will assess each study across these domains, and any discrepancies will be resolved by involving a third reviewer. The overall risk of bias for each study will be determined based on the “worst-score rule,” meaning that the final judgment reflects the highest level of bias identified in any single domain.

In addition to ROBINS-I, other tools may be considered based on study design. For cohort and case-control studies, the Newcastle-Ottawa Scale may be used, which judges study selection, comparability, outcome and exposure domains via a star system, and classifies overall quality (good/fair/poor) using established thresholds [25]. Diagnostic accuracy or method-comparison studies (eg, assay sensitivity and specificity against a reference standard) will be appraised with QUADAS-2 (Quality Assessment of Diagnostic Accuracy Studies tool), covering the case selection, index test, reference standard, and flow/timing that led to domain-level ratings (low, high, or unclear) and an overall judgment [26]. For analytical validation without a case-level reference, we will apply adapted QUADAS-2 signaling questions focused on sample handling, analyst blinding, repeatability, and selective reporting. In studies with overlapping designs or mixed components such as those reporting both exposure-outcome associations and diagnostic accuracy, we will apply more than one appraisal tool as appropriate to each component. In such cases, we will present risk-of-bias ratings separately for each tool and component. If this yields differing overall judgments, we will prioritize the tool corresponding to the study’s primary aim or default to the higher risk rating for synthesis purposes. The rationale for tool selection and integration will be documented. However, it should be noted that these alternative tools are generally less comprehensive than the ROBINS-I tool, particularly when adapted for assessing bias in exposure studies. A quality summary will be presented in a table or figure.

Two independent reviewers will complete domain-level ratings using prespecified guidance and standardized forms; disagreements will be resolved by discussion and, if necessary, a third reviewer, defaulting to the more high-risk rating when consensus is not achievable. We will document rationales for each judgment and present domain- and study-level summaries in tables or traffic-light plots.

### Statistical Analysis

Statistical analysis will be performed using Review Manager (RevMan; version 5.4; The Cochrane Collaboration). A meta-analysis will be conducted if at least two comparable studies with similar outcome measures are available. Meta-analyses will be conducted for viral load quantifications from included articles to determine overall effect sizes. Effect sizes will be calculated as risk ratios or odds ratios for categorical outcomes (eg, detection of DENV in wastewater) and as mean differences or standardized mean differences for continuous outcomes (eg, viral load or concentrations of dengue markers) [27]. Heterogeneity will be assessed using the *I*² statistic and Q test. The *I*² statistic will be used to quantify the proportion of total variation across studies attributable to heterogeneity rather than chance, with thresholds of 25%, 50%, and 75% representing low, moderate, and high heterogeneity, respectively [28]. The Q test, which will apply a *χ*^2^ test, will be used to statistically evaluate heterogeneity among the studies.

Pooled analyses that use fixed-effects models for low heterogeneity (*I*²<50%) and random-effects models for moderate to high heterogeneity (*I*²≥50%), accounting for variability across studies, will be performed [29]. Subgroup analyses will be performed based on factors such as sampling techniques, detection methods, dengue serotypes, sampling sites, geographic location, and seasonality. Sensitivity analyses will be conducted to assess the robustness of the overall findings by excluding studies with high risk of bias or outlying results [30]. Cumulative meta-analysis will track trends in effect sizes over time [31]. Studies meeting inclusion criteria but lacking data for meta-analysis will be summarized narratively and compared with the meta-analysis findings.

### Certainty of Evidence

The certainty of evidence will be systematically evaluated using the GRADE (Grading of Recommendations Assessment, Development, and Evaluation) framework [32,33]. This evaluation will address (1) study limitations, considering potential biases in selection, performance, detection, and reporting that may affect the reliability of the results; (2) inconsistency, where variations in findings across studies may undermine confidence in the estimated effect size; (3) indirectness, which will assess the applicability of the evidence to the research question by examining differences in populations, interventions, and outcomes across studies; (4) imprecision, which will evaluate the certainty of the effect estimates, with wide confidence intervals reflecting uncertainty in the findings; and (5) publication bias [33]. Each of these domains will be rated to determine the overall quality of the evidence as high, moderate, low, or very low.

### Assessment of Reporting Biases

Publication bias will be assessed using a funnel plot by visualizing the effect sizes of different studies, particularly between small and large study sizes. Further tests including the Egger regression test significance at *P*<.05 [34] will be suggested if bias is suspected, given the number of articles included will be more than 10 [30].

## Results

**Figure 1.**
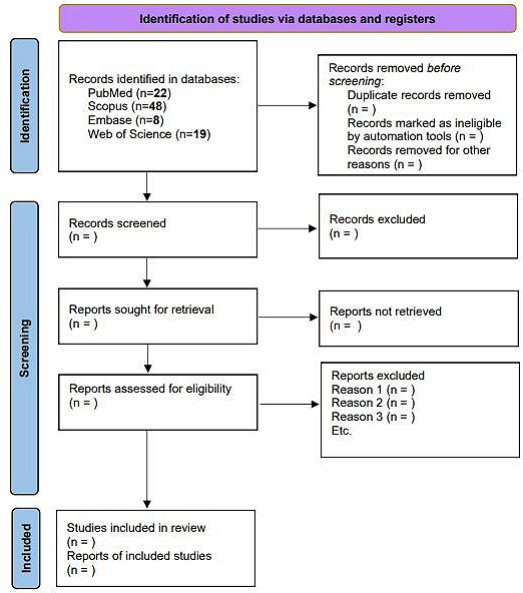
PRISMA flow diagram. PRISMA: Preferred Reporting Items for Systematic Reviews and Meta-Analyses.

A PRISMA 2020 flow diagram will be presented to illustrate the process of study identification, screening, and final inclusion for this systematic review and meta-analysis (Figure 1) [35]. Results will be charted and tabulated to provide a detailed summary of methodologies and outcomes. The study characteristics and key outcomes including dengue WBS methods, detection rates of the DENV, viral load, and the accuracy of these methods in terms of sensitivity and specificity will be presented. The meta-analysis of viral load quantification and subgroup analysis will be visualized using forest plots. As of October 2025, records have been identified from the databases and data analysis is expected to be completed by the first quarter of 2026.

## Discussion

### Expected Outcomes

This systematic review and meta-analysis will describe and synthesize the current evidence of dengue WBS in terms of the available methodologies and their correlation with viral concentration. The reviewed studies are expected to demonstrate the detection and quantification of DENV in population-based WBS and dengue-spiked experimental setups. Importantly, these studies will inform the selection of critical methodologies for conducting dengue WBS, including sampling strategies, sample types, concentration methods, nucleic acid extraction, and detection techniques. Additionally, they will offer insights into the feasibility and practical challenges of implementing WBS for dengue surveillance.

### Strengths and Limitations

This will be the first comprehensive review on the global application of this surveillance method for dengue. WBS has been increasingly recognized as a cost effective and non-invasive tool to monitor infectious diseases in the community setting and its application has great potential to enhance public health measures and improve disease outcomes [36]. The data from the WBS could complement clinical surveillance to predict possible dengue outbreaks and provide an early warning through the detection of asymptomatic cases. This review will also guide the selection of key methodologies in performing dengue WBS and identify the gaps and limitations of different sampling and detection methods.

Given the emerging status of this field, we anticipate a limited number of studies will be available, which could limit the comparability and meta-analysis of the sampling and detection methods. There is also a possibility of reporting biases, with a higher frequency of positive results being published than negative findings.

## Supplementary material

10.2196/71635 Checklist 1 PRISMA-P checklist.

## Abbreviations

DENV: dengue virus
GRADE: Grading of Recommendations Assessment, Development, and Evaluation
MeSH: Medical Subject Headings
PRISMA: Preferred Reporting Items for Systematic Reviews and Meta-Analyses
PRISMA-P: Preferred Reporting Items for Systematic Review and Meta-Analysis Protocols
PROSPERO: International Prospective Register of Systematic Reviews
QUADAS: Quality Assessment of Diagnostic Accuracy Studies
WBS: wastewater-based surveillance
